# The gp38 protein inhibits host adsorption of phage vB_EcoM_SD286

**DOI:** 10.3389/fmicb.2026.1751343

**Published:** 2026-02-13

**Authors:** Xiaojing Lei, Xueling Wang, Haoyue Shang, Tingting Zhang, Jiangtao Xu, Liang Zhang, Duanduan Chen, Guodong Zhou, Yubao Li, Zhenshu Si, Shengliang Cao

**Affiliations:** 1 College of Agriculture and Biology, Liaocheng University, Liaocheng, Shandong, China; 2 Shandong Fengxiang (Group) Co., Liaocheng, Shandong, China; 3 School of Pharmacy and Food Engineering, Liaocheng University, Liaocheng, Shandong, China

**Keywords:** adsorption inhibition, biological characteristics, *Escherichia coli* phage vB_EcoM_SD286, genetic analysis, protein gp38

## Abstract

**Background:**

Bacteriophages (phages), which are viruses that infect bacteria, primarily use surface receptor-binding proteins (RBPs) to recognize and infect their hosts. Elucidating the function of specific RBPs is crucial for understanding phage-host interactions and developing phage-based antimicrobials.

**Methods:**

This study characterized the *Escherichia coli* phage vB_EcoM_SD286, isolated from farm sewage in Shandong Province. Its morphology was observed via transmission electron microscopy. The lysis spectrum and optimal multiplicity of infection (MOI) were determined using the double-layer plate method. Stability under various pH and temperature conditions was assessed. A one-step growth curve was plotted to determine the latent period and burst size. The genome was sequenced and analyzed for open reading frames (ORFs), tRNA, virulence factors, and antibiotic resistance genes. Bioinformatic analysis suggested that the putative protein gp38 may function as an RBP. To verify this, a recombinant expression vector, pET-28a(+)-gp38, was constructed and induced in BL21(DE3) cells to produce the recombinant gp38 protein. Competitive adsorption and binding assays were conducted to evaluate its role in host recognition.

**Results:**

Phage vB_EcoM_SD286 exhibited an icosahedral head and a helical tail, classifying it within the *Caudoviricetes* class and *Rosemountvirus* genus. It lysed 39% of tested strains, with an optimal MOI of 0.01. The phage demonstrated stability across a broad pH range (4–12) and at temperatures below 50°C, but was completely inactivated after 20 min at 80°C. The one-step growth curve revealed a 25 min latency period and a burst size of 33 PFU/cell. Whole-genome sequencing revealed a 52,891 bp genome with 46.06% GC content, containing 74 ORFs but no tRNAs, virulence factors, or antibiotic resistance genes. The recombinant gp38 protein was successfully expressed. Subsequent competitive adsorption assays, alongside direct binding experiments between host bacteria and the gp38 protein, demonstrated that gp38 significantly inhibited phage adsorption to host bacteria.

**Conclusion:**

Collectively, our findings provide preliminary evidence that gp38 is involved in the phage-host interaction of vB_EcoM_SD286, likely functioning as a receptor-binding protein. This study offers a theoretical basis for elucidating the precise bacterial receptor recognition mechanism and lays the groundwork for future development of phage-based antimicrobial agents.

## 1 Introduction

Avian colibacillosis, primarily caused by avian pathogenic *Escherichia coli* (APEC), is a prevalent and economically significant disease in poultry. It leads to acute septicemia, airsacculitis, pericarditis, perihepatitis, and other lesions, which severely impair growth performance and egg production, resulting in substantial economic losses (Hu et al., 2022; Nawaz et al., 2024). APEC exhibits high mutability, multiple serotypes, and considerable antigenic variation among serotypes, which hinders vaccine development and application (Runcharoon et al., 2025). Currently, combination antibiotic therapy is the most common approach for controlling avian colibacillosis (Iwu et al., 2020; Tohmaz et al., 2022). However, the indiscriminate and inappropriate use of antibiotics has promoted multidrug-resistant bacteria, leading to reduced antibiotic efficacy, public health risks, and food safety concerns due to drug residues (Tohmaz et al., 2022; Aworh et al., 2025; Risalvato et al., 2025). Therefore, developing alternative antimicrobial strategies has become urgent, among which phage therapy has gained significant attention (Yao et al., 2023; Karami et al., 2024).

Bacteriophages (phages) are viruses that infect and lyse host bacteria (Strathdee et al., 2023). Compared with antibiotics, phage preparations offer several advantages. They exhibit strong host specificity, can target multidrug-resistant bacteria, do not disrupt intestinal microbiota balance, and are non-toxic (Rodriguez-Gonzalez et al., 2020; Egido et al., 2022; Uyttebroek et al., 2022). However, a narrow host range remains the main challenge limiting phage therapy. Phage adsorption, the critical first step in infection, involves specific binding of surface receptor-binding proteins (RBPs) to bacterial receptors, which determines infection specificity (Ding et al., 2024). RBPs and their corresponding receptors differ among phages in both structure and specificity (Ning et al., 2024). Before infection, an *E. coli* phage must recognize and attach to specific host receptors through its RBPs (Chen et al., 2020). These proteins are typically located in the tail region, such as the tail fibers and tail spikes of T4 phage, and specifically bind to surface receptors of E. *coli*, including lipopolysaccharides and outer membrane proteins (Suga et al., 2021). For instance, the T4 phage recognizes the OmpC protein on the bacterial surface through the gp37 protein located at the tip of its tail fiber (Brzozowska et al., 2015). During early infection, the tail fibers and spikes of T4 undergo conformational changes that trigger tail sheath contraction, enabling the tail tube to penetrate the bacterial membrane (Wenzel et al., 2024). This highly specific molecular recognition mechanism restricts the T4 phage to infecting only specific *E. coli* strains. However, the potential role of other unknown proteins in the early stages of phage adsorption remains insufficiently explored.

This study aimed to isolate and characterize the novel bacteriophage vB_EcoM_SD286, identify its key functional receptor-binding protein through genomic and bioinformatic analyses, and experimentally validate the adsorption activity of the candidate protein gp38. By confirming the receptor-binding protein (RBP)-like function of gp38, this work establishes a methodological framework for the identification of novel RBPs in *Escherichia coli* phages. Furthermore, it provides molecular targets and a theoretical foundation for developing engineered broad-spectrum phage therapies, as well as novel antimicrobial or diagnostic tools, with potential application against infections caused by bacterial pathogens such as APEC.

## 2 Materials and methods

### 2.1 Bacterial strains and growth conditions

*Escherichia coli* SD405 and 100 additional clinical isolates of *E. coli* were obtained from the Phage Research Center Laboratory, Liaocheng University. Each strain was streaked onto MacConkey agar plates for activation after retrieval from the strain bank and incubated inverted at 37°C overnight. A single colony from each plate was inoculated into 5 mL of LB broth in centrifuge tubes and incubated at 37°C and 200 rpm for 8–12 h to ensure active growth.

### 2.2 Phage isolation and purification

The isolation and purification of the phage was tested using a modified version of the method described by Abdelrahman et al. (2022). A 200 μL sample of sewage-derived phage was added to a centrifuge tube containing 5 mL of LB broth and 200 μL of the corresponding *E. coli* host culture. The mixture was incubated at 37°C and 220 rpm for 4 h. After incubation, the culture was centrifuged at 5000 rpm for 5 min, and the supernatant was filtered through a 0.22 μm membrane to obtain the fresh phage lysate. For purification, a single plaque is picked using a sterile pipette tip, transferred into SM buffer, and vortexed thoroughly. The mixture is then filtered through a 0.22 μm filter. The filtrate is combined with host bacteria to prepare double-layer plates again. The process of picking a single plaque is repeated for 4–6 times to obtain purified phage.

### 2.3 Phage titer and transmission electron microscopy

A high-titer phage lysate was prepared using the double-layer agar amplification method. A logarithmic-phase bacterial culture was mixed with the phage at the optimal multiplicity of infection (MOI) and incubated at 37°C in a water bath for 20 min. Luria-Bertani (LB) semi-solid agar (approximately 50°C) was added, mixed, and poured onto LB solid agar plates. After solidification, the plates were inverted and incubated at 37°C for 8–10 h. Once phage plaques fully developed, 3–5 mL of SM buffer was added to the plate, followed by shaking at 100 rpm for 4 h. The eluate was collected and clarified by low-speed centrifugation (8,000 × g, 10 min, 4°C) to remove bacterial debris. The buffer was then filtered through a 0.22 μm membrane.

For titer determination, 10-fold serial dilutions (e.g., 10^–1^ to 10^−8^) of the lysate were prepared in LB broth. A 100 μL aliquot of each dilution was mixed with 100 μL of a fresh logarithmic-phase host culture, to make a final mixture of 200 μL. This mixture was then blended with LB semi-solid agar (55°C), and immediately overlaid onto LB plates. After overnight incubation at 37°C, plaques were counted. The phage titer (PFU/mL) was calculated using the formula: Titer = (number of plaques) × (dilution factor) × 10.

For morphological observation by transmission electron microscopy (TEM), phage particles were concentrated and purified. The filtered lysate was treated with polyethylene glycol 8000 (PEG 8000) at a final concentration of 10% (w/v) and incubated at 4 °C for 1 h to overnight. The mixture was then centrifuged at 12,000 × g for 30 min at 4 °C. The resulting pellet was resuspended in a minimal volume of SM buffer. When higher purity was required, an additional purification step using CsCl density gradient centrifugation or ultrafiltration concentrators was performed. The concentrated phage preparation was applied to carbon-coated copper grids, negatively stained with 2% (w/v) phosphotungstic acid (pH 7.0), and examined using transmission electron microscopy (TEM) (Li et al., 2022).

### 2.4 Determination of phage lysis profile and efficiency of plating

To determine the phage Lysis Profile, we utilized the method described by Kizheva et al. (2025). The host range of the phage was determined using the double-layer agar drop method. A 5 mL aliquot of LB semi-solid agar (approximately 55°C) was poured into each of multiple 10 mL centrifuge tubes containing 100 mL of different *E. coli* cultures, mixed thoroughly, and poured onto a pre-prepared LB agar plate, followed by gentle shaking for even distribution. Then, 5 μL of phage lysate was dropped onto the solidified surface and allowed to stand for 10 min before incubation at 37°C for 8–10 h. The formation of clear plaques indicated that the phage could lyse the bacterial strain, whereas no plaque formation indicated the absence of lytic activity. Some strain information was consistent with previously published reports from our laboratory (Huang et al., 2025).

For bacterial strains that tested positive in the spot assay, productive infection was further assessed by determining the efficiency of plating (EOP) (Campos et al., 2025). Overnight cultures of each test strain and the primary host strain, *E. coli* SD405, were freshly grown in LB broth at 37 °C for 12 h. Phage lysates were serially diluted (10^–3^ to 10^−7^), mixed with different host bacteria, and titers were determined using a standard double-layer agar plaque assay, followed by overnight incubation at 37 °C. All assays were performed in triplicate.

The EOP was calculated as the mean plaque-forming units (PFU) on a test strain divided by the mean PFU on the primary host, *E. coli* SD405. An EOP value ≥ 0.5 was considered to indicate high production efficiency, reflecting strong replication in the test host. Values between 0.1 and 0.5 were categorized as medium efficiency, corresponding to a moderate level of productive infection.

### 2.5 Optimal multiplicity of infection of phage

To determine the optimal multiplicity of infection (MOI) of the bacteriophages, we utilized the method described by Li et al. (2019), The MOI, defined as the ratio of phage particles to host bacterial cells, was determined as follows. The host bacterial suspension was adjusted to 10^8^ CFU/mL, and phage suspensions were adjusted to 10^8^, 10^7^, 10^6^, and 10^5^ PFU/mL. Equal volumes of host and phage suspensions were mixed to achieve MOI values of 10, 1, 0.1, 0.01, and 0.001, respectively. The mixtures were incubated at 37°C in a shaking incubator for 3 h, followed by centrifugation at 12,000 rpm for 2 min and filtration through a 0.22 μm membrane. The resulting phage lysates were serially diluted (10-fold), and the phage titer was determined using the double-layer agar method. The MOI yielding the highest titer was recorded as the optimal MOI for that phage.

### 2.6 pH stability of phage

The stability of PH was determined using a modification of previously described methods (Wu et al., 2025). The pH stability of the phage was assessed by adjusting LB broth to pH 1–14 using hydrochloric acid or sodium hydroxide. A phage lysate with a titer of 4.74 × 10^8^ PFU/mL was used for the assay. Specifically, 100 μL of this phage lysate was mixed with 900 μL of LB broth at the designated pH and incubated at 37°C for 1, 2, and 3 h, respectively. The mixture was then centrifuged at 12,000 rpm for 5 min, filtered through a 0.22 μm membrane, and serially diluted (10-fold) for titer determination using the double-layer agar method. Each experiment was performed in triplicate to ensure reproducibility.

### 2.7 Chloroform stability of the phage

The chloroform stability of the phage was tested using a modified version of the method described by Gao et al. (2023). The phage suspension used had a titer of 3.1 × 10^8^ PFU/mL. Phage suspensions (1.0, 0.9, 0.8, 0.7, 0.6, and 0.5 mL) were added to 1.5 mL microcentrifuge tubes, and chloroform solutions (0, 10, 20, 30, 40, and 50%) were added to reach a final volume of 1 mL. The samples were mixed thoroughly and incubated at 37°C for 30 min, 1 h, and 2 h, respectively. The mixtures were then centrifuged at 12,000 rpm for 10 min. Aliquots (100 μL) from each tube were serially diluted (10-fold), and the phage titer was determined using the double-layer agar method. Each test was performed in triplicate.

### 2.8 Temperature stability of the phage

The stability of temperature was determined using a modification of previously described methods (Cao et al., 2023). A phage lysate with a titer of 5.2 × 10^8^ PFU/mL was used for the assay. To evaluate thermal stability, 500 μL of phage lysate was incubated in a water bath at 4, 25, 40, 50, 60, 70, and 80°C. Samples were collected at 20, 40, and 60 min for each temperature. The lysates were serially diluted (10-fold), and phage titers were determined using the double-layer agar method. Each experiment was conducted in triplicate to ensure reproducibility.

### 2.9 One-step growth curve of the phage

To determine the one-step growth curve of the bacteriophages, we utilized the method described by Wan et al. (2025), A phage lysate with a titer of 1.5 × 10^8^ PFU/mL was used. The one-step growth curve was determined by mixing the host bacterial culture and phage lysate at the optimal MOI and incubating at 37°C for 5 min. The mixture was centrifuged at 12,000 rpm for 5 min, and the supernatant was discarded. The pellet was washed twice with preheated LB broth (37°C), resuspended in 1 mL of preheated LB broth, and transferred to 100 mL of preheated LB medium. Incubation was carried out at 37°C for 120 min in a constant-temperature shaker. Beginning at 0 min, samples were collected every 10 min, filtered through a 0.22 μm membrane, and used to obtain phage lysates. After 10-fold serial dilution, titers were determined using the double-layer agar method. The burst size (PFU/cell) was calculated using the following equation: Burst size = (Final phage titer – Initial phage titer)/Initial number of infected host bacteria. All experiments were performed in triplicate.

### 2.10 Phage genome sequencing and comparative genomics analysis

Phage DNA was extracted using the E.Z.N.A.^®^ DNA Kit (OMEGA, United States). Sequencing libraries were prepared using the TruSeq™ Nano DNA Sample Prep Kit (Illumina, United States), and whole-genome sequencing was conducted on the Illumina NovaSeq 6000 platform by Shanghai Lingen Biotechnology Co., Ltd. (Shanghai, China). Raw data quality control was performed using Trimmomatic v0.36 (Bolger et al., 2014), and ABySS v2.2.0 was used for genome assembly. Protein-coding genes were predicted using GeneMarkS v4.17 (Besemer and Borodovsky, 2005), a tool optimized for viral genomes. The predicted gene products were functionally annotated through BLASTp searches against the NCBI non-redundant protein database (NR) to assign putative functions. Functional classification information was further retrieved from the COG, GO, and KEGG databases based on the BLASTp results. Protein domain analysis (e.g., Pfam) and identification of specific enzyme families (e.g., CAZy) were similarly performed using information derived from these BLASTp alignments. Sequence similarity was assessed via BLASTp searches against the NCBI database^1^ (Altschul et al., 1997). tRNA genes were predicted using tRNAscan-SE v2.0^2^ (Lowe and Chan, 2016). Virulence and antibiotic resistance genes were identified using the Virulence Factor Database^3^ and the Comprehensive Antibiotic Resistance Database (CARD)^4^ (Cao et al., 2023). The lifestyle of phage vB_EcoM_SD286 was computationally predicted using the bioinformatics tool PhaBOX (Shang et al., 2022). The complete genome sequence of the phage was submitted to GenBank, and BLASTn was used to compare phage vB_EcoM_SD286 with sequences available in the database. Phage tBLASTx analysis was conducted via the Virus Proteome Tree (Viptree) server^5^ (Nishimura et al., 2017) to construct a phage proteome tree based on whole-genome similarity (SG). Phylogenetic analysis was performed using the VICTOR web service^6^ (Meier-Kolthoff and Göker, 2017), a method commonly used for phage genome-based phylogeny and classification. Based on the NCBI BLASTn results, 15 homologous sequences with the highest similarity were selected. Pairwise genomic distances and similarities were calculated using the VIRIDIC platform^7^ (Moraru et al., 2020).

### 2.11 Basic physicochemical properties and structural analysis of the gp38 protein

Based on the amino acid sequence of the gp38 protein, its basic physicochemical characteristics were analyzed using the ProtParam platform^8^ (Gasteiger et al., 2003). The hydrophilicity and hydrophobicity of gp38 were evaluated using ProtScale.^9^ Transmembrane domain prediction was performed using the TMHMM Server v2.0^10^ (Santos Junior et al., 2020), and signal peptide analysis was conducted using NovoPro SignalP^11^ (Almagro Armenteros et al., 2019). The secondary and tertiary structures of gp38 were predicted using PSIPRED^12^ (Buchan et al., 2024) and the SWISS-MODEL homology modeling server^13^ (Waterhouse et al., 2018), respectively. Furthermore, the three-dimensional structures of the three proteins relevant to the subsequent experiments were predicted using AlphaFold3^14^ (Jumper et al., 2021).

### 2.12 Construction of the recombinant plasmid

Construction of the recombinant plasmid was performed using a modified version of the method described by Azizoglu et al. (2016). Primers targeting the gp38 gene of phage vB_EcoM_SD286 were designed using SnapGene software, based on the previously sequenced genome. Restriction enzyme sites *Bam*HI and *Xho*I were incorporated into the primers. The forward primer 5’-CGGGATCCATGGCACAAGCGAAAGCTGACC-3’, and reverse primer 5’-CCCTCGAGTTTACTCGGCTTCTTCCGTAGCT-3’ were synthesized by Sangon Biotech (Shanghai, China). Using the designed gp38-F/R primers and phage DNA as the template, Polymerase Chain Reaction (PCR) amplification was performed to obtain the target fragment. The PCR amplification was performed under the following conditions: Initial denaturation at 95°C for 5 min, followed by 30 cycles at 94°C for 1 min, 67.9°C for 1 min, 72°C for 0.5 min, and a final extension step at 72°C for 10 min. Both the pET-28a(+) vector and the gp38 insert were digested with *Bam*HI and *Xho*I (Takara Bio Inc.), followed by ligation at 16°C overnight using T4 DNA ligase (Takara Bio Inc.). The recombinant constructs were verified by colony PCR and double-enzyme digestion. Positive colonies were confirmed by Sanger sequencing, and successfully constructed recombinant plasmids pET-28a(+)-gp38 were stored at −80°C for future use.

Following an identical experimental procedure, recombinant plasmids for the control proteins were constructed. The primers used were as follows:

For gp66 (GenBank: YAO58176.1): Forward 5’-CGGGATC CATGTATATGGCTGATGTAC-3’, Reverse 5’-CCAAGCTTG TTACGATACGTTGCTTACG-3’; For lysozyme (lys) (GenBank: YP_009845677.1): Forward 5’-ATGAAAGTGCAAT ACAAAGATATTGAT-3’, Reverse 5’-CTATCGGGTACGTTCT AAAACG-3’.

Appropriate annealing temperatures and extension times were optimized for each protein respectively. The resulting recombinant plasmids were stored at −80°C.

### 2.13 Protein expression and purification

The recombinant plasmid pET-28a(+)-gp38 was transformed into *E. coli* BL21(DE3) competent cells (Sangon Biotech) to generate the recombinant strain BL21-pET-28a(+)-gp38. The same transformation procedure was followed for the expression of the gp66 and lys proteins. An overnight culture of the recombinant strain was inoculated at a 1:100 ratio into LB broth supplemented with kanamycin (Solarbio) and incubated until the logarithmic phase (OD_600_ = 0.4–0.6). Isopropyl β-D-Thiogalactopyranoside (IPTG) was added to a final concentration of 0.5 mM, and induction was carried out for 5 h. The culture was centrifuged at 4°C and 12,000 rpm for 10 min, and the cell pellet was collected. The cells were washed twice with phosphate-buffered saline (PBS), sonicated at 4°C, and centrifuged again at 4°C and 12,000 rpm for 15 min. The supernatant and pellet were collected separately, and protein expression in both fractions was analyzed using sodium dodecyl sulfate–polyacrylamide gel electrophoresis (SDS–PAGE).

The SDS-PAGE gels were prepared using a 12.5% ExpressCast PAGE precast gel kit. After cleaning and assembling the glass plates, the separating gel was prepared according to the instructions, poured to an appropriate height, and overlaid with water for 20 min. Following the removal of the water layer and drying, the stacking gel was added, and a comb was inserted until complete polymerization. The polymerized gel was installed in the electrophoresis tank and immersed in running buffer. The boiled protein samples were cooled to room temperature prior to loading, with 10 μL of sample loaded per well alongside 5 μL of protein marker as a reference. Electrophoresis was initially performed at 80 V until the dye front entered the separating gel(typically about 20 min), after which the voltage was increased to 120 V and maintained until the dye front reached the bottom of the gel. Upon completion, the gel was removed and stained with Coomassie Brilliant Blue for 2–4 h (or overnight), followed by destaining until clear protein bands were visible. Finally, the gel was photographed and analyzed.

For purification, nickel affinity chromatography was performed to isolate inducibly expressed recombinant proteins. The inclusion bodies were dissolved in column equilibration buffer (100 mM NaH_2_PO_4_, 10 mM Tris-Cl, 8 M urea, pH 8.0) and incubated at room temperature for 30–60 min. The mixture was then centrifuged at 12,000 rpm for 30 min to remove insoluble material. A nickel–agarose column was pre-equilibrated by rinsing with five column volumes of deionized water, followed by five column volumes of equilibration buffer. The supernatant was loaded onto the column, and unbound material was washed off with five column volumes of washing buffer (100 mM NaH_2_PO_4_, 10 mM Tris-Cl, 10 mM imidazole, 8 M urea, pH 8.0). The target protein was then eluted using five column volumes of elution buffer (100 mM NaH_2_PO_4_, 10 mM Tris-Cl, 250 mM imidazole, 8 M urea, pH 8.0). Refolding of inclusion body proteins was performed following previously reported methods (Ni et al., 2016; Ye et al., 2019). Protein purity across the elution fractions was assessed by SDS–PAGE, and the molecular size was reconfirmed. The protein concentration was determined using the Bradford Protein Assay Kit (Beyotime Biotechnology). The purified recombinant gp38 protein was stored at −80°C until further use.

### 2.14 Verification of the competitive adsorption function of gp38 protein in vB_EcoM_SD286

The effect of the gp38 protein on phage adsorption was verified through competitive adsorption assays using *E. coli* SD405 (10^8^ CFU/mL) and phage vB_EcoM_SD286 (10^8^ PFU/mL). The optimal MOI for vB_EcoM_SD286 was 0.01, and the incubation period was 20 min. The procedure was adapted from previously reported methods (Ge et al., 2023), as described below. Group 1: *E. coli* SD405 (1 mL) was mixed with 10 μL of vB_EcoM_SD286 and incubated at 37°C in a shaker for 15 min. Group 2: *E. coli* SD405 (1 mL) was mixed with 140 μL of gp38 protein (1.6 mg/mL) and incubated at 37°C for 20 min, followed by the addition of 10 μL of vB_EcoM_SD286 and further incubation for 15 min under the same conditions. Group 3: SM buffer (1 mL) was mixed with 10 μL of vB_EcoM_SD286 and incubated at 37°C for 15 min. Group 4: *E. coli* SD405 (1 mL) was mixed with 70 μL of gp38 protein (0.8 mg/mL) and incubated at 37°C for 20 min. Then, 10 μL of vB_EcoM_SD286 was added, mixed thoroughly, and incubated at 37°C for 15 min. LB broth was added to equalize the final volume with that of Group 2. After incubation, all mixtures were centrifuged at 4°C and 12,000 rpm for 10 min. The supernatants were collected, and the titers of free vB_EcoM_SD286 were determined using the double-layer agar method. A negative control was included using an unrelated protein that neither affected phage adsorption nor bound to the host bacterium, and a positive control was established using gp66, which shares homology with the tail filament protein gp41 previously reported in vB_EcoM_SD350 (Huang et al., 2025).

To assess potential bacteriolytic activity of the proteins, bacterial viability was evaluated. *E. coli* SD405 (1 mL at 10^8^ CFU/mL) was incubated with each test protein—gp38, gp66, or Lys—at a final concentration of 1.6 mg/mL, along with a control treated with an equal volume of LB broth. All samples were incubated at 37 °C for 20 min, with consistent final reaction volumes across groups. Following incubation, the mixtures were serially diluted 10-fold in sterile LB broth, and 100 μL of each dilution was plated onto LB agar. Plates were incubated overnight at 37 °C, and bacterial concentration was determined by colony counting (CFU/mL). Each test was performed in triplicate.

### 2.15 Host bacterium and gp38 protein binding experiment

This experiment was conducted with modifications based on established methods (Ding et al., 2024). Host bacteria (100 μL) were inoculated into 5 mL of LB broth and incubated at 37°C and 220 rpm until reaching an OD_600_ of 0.3 (approximately 10^8^ CFU/mL). The culture (5 mL) was transferred to a 10 mL centrifuge tube, centrifuged at 8,000 rpm for 10 min at room temperature, and the supernatant was discarded. The bacterial pellet was washed three times with 1 mL of 1 × PBS and resuspended in 1 × PBS containing gp38 protein to yield a final concentration of 10^8^ CFU/mL. The bacteria–protein mixture was incubated for 0.2, 0.5, 2, 4, 8, 12, 16, and 20 min, followed by rapid centrifugation at 4°C and 12,000 × g for 30 s. The supernatant was collected, and protein concentration was determined using a BCA protein assay kit. A negative control consisted of protein solution not incubated with bacteria, and a positive control consisted of bovine serum albumin (BSA, 1.5 mg/mL) incubated with host bacteria.

### 2.16 Statistical analysis

All data were analyzed using GraphPad Prism v8.4.3. Statistical significance was evaluated by multiple *t*-tests, and all experiments were performed in triplicate. Results are presented as mean ± standard deviation (SD) (Wen et al., 2024).

## 3 Results

### 3.1 Morphological characterization, host Range, and efficiency of plating of the phage vB_EcoM_SD286

**FIGURE 1:**
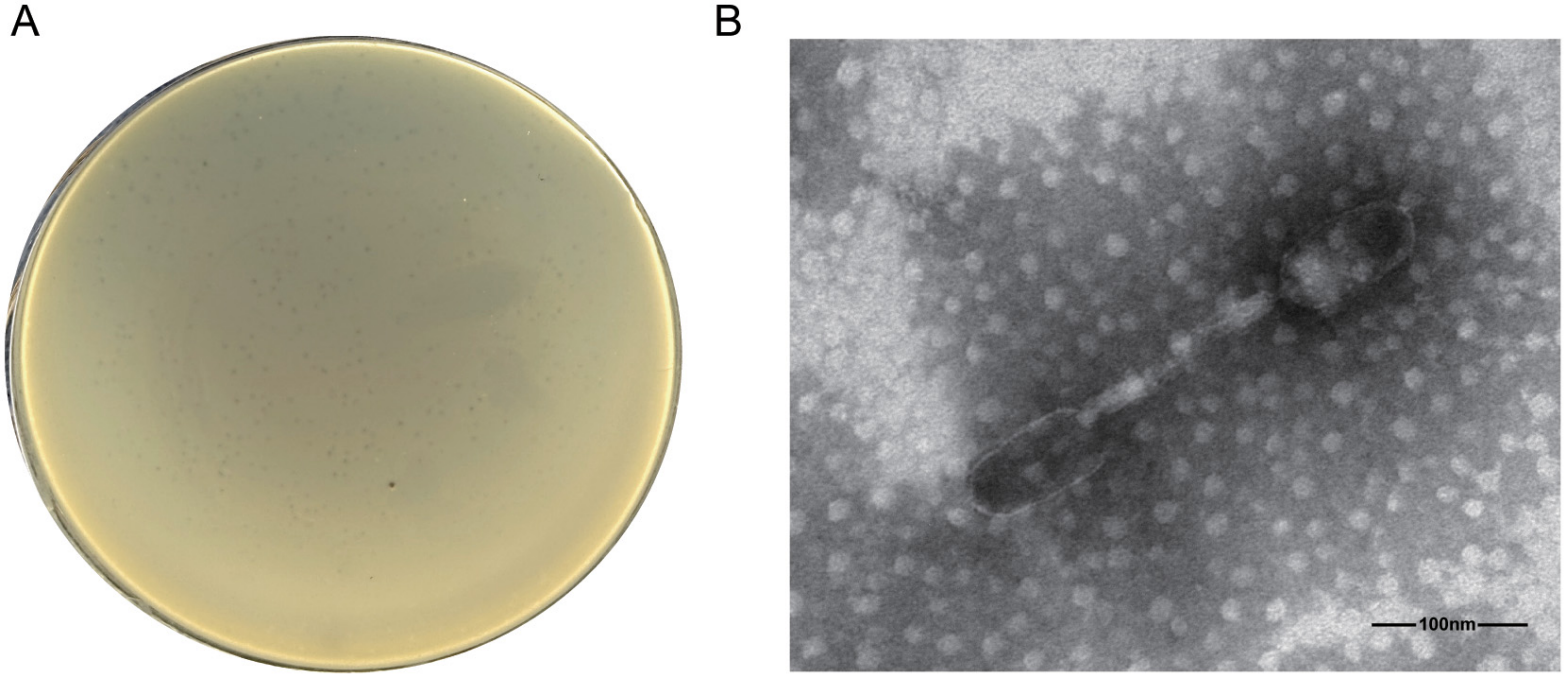
Phage vB_EcoM_SD286 isolation, identification, and morphological observation. **(A)** The purified phage vB_EcoM_SD286 exhibits uniformly sized plaques. **(B)** Transmission electron microscope morphology of phage vB_EcoM_SD286. Scale bar = 100 nm.

Phage vB_EcoM_SD286 was isolated from sewage at a farm in Shandong Province and underwent six rounds of purification. Using the double-layer agar plate method, the titer of vB_EcoM_SD286 was 1.5 × 10^8^ PFU/mL, producing uniformly sized phage plaques (Figure 1A). TEM revealed that vB_EcoM_SD286 belongs to the tailed phage order, muscle-tailed phage family, with a head shaped like a regular icosahedron and a tail exhibiting helical symmetry (Figure 1B). *E. coli* strains isolated from different regions between 2018 and 2024 by our laboratory were used to determine the host range of vB_EcoM_SD286 through the double-layer plate spot method. The phage was found to infect 39 *E. coli* strains, showing a coverage rate of 39% (Table 1).

**TABLE 1:** Lytic activity of phage vB_EcoM_SD286 against *Escherichia coli.*

No.	Bacterial strain name	Bacterial strain	Separation year	Place	Source	Lytic ability	Efficiency of plating
1	SD164	*E. coli*	2020	Weifang	Chicken	+	R
2	SD166	*E. coli*	2020	Weifang	Chicken	+	R
3	SD169	*E. coli*	2019	Weifang	Chicken	+	R
4	SD170	*E. coli*	2018	Jinan	Chicken	+	R
5	SD179	*E. coli*	2018	Jinan	Chicken	+	R
6	SD197	*E. coli*	2019	Jinan	Chicken	+	R
7	SD199	*E. coli*	2019	Jinan	Chicken	+	R
8	SD281	*E. coli*	2019	Dezhou	Chicken	+	R
9	SD285	*E. coli*	2019	Dezhou	Chicken	+	R
10	SD288	*E. coli*	2019	Yantai	Duck	+	0.85
11	SD317	*E. coli*	2020	Taian	Chicken	+	R
12	SD319	*E. coli*	2020	Taian	Chicken	+	R
13	SD320	*E. coli*	2020	Taian	Chicken	+	R
14	SD321	*E. coli*	2020	Taian	Chicken	+	R
15	SD322	*E. coli*	2020	Taian	Chicken	+	R
16	SD325	*E. coli*	2020	Taian	Chicken	+	R
17	SD326	*E. coli*	2020	Taian	Chicken	+	R
18	SD327	*E. coli*	2020	Taian	Chicken	+	R
19	SD374	*E. coli*	2021	Yantai	Chicken	+	R
20	SD375	*E. coli*	2020	Weifang	Chicken	+	R
21	SD387	*E. coli*	2020	Weifang	Chicken	+	R
22	SD392	*E. coli*	2019	Dongying	Chicken	+	R
23	SD399	*E. coli*	2021	Yantai	Chicken	+	R
24	SD405	*E. coli*	2020	Weifang	Chicken	+	1
25	SD430	*E. coli*	2020	Liaocheng	Chicken	+	R
26	SD434	*E. coli*	2021	Yantai	Chicken	+	R
27	SD323	*E. coli*	2019	Dongying	Chicken	+	R
28	SD324	*E. coli*	2019	Dongying	Chicken	+	0.5
29	SD350	*E. coli*	2019	Dongying	Chicken	+	0.98
30	SD339	*E. coli*	2019	Dongying	Chicken	+	0.33
31	SD426	*E. coli*	2020	Liaocheng	Chicken	+	R
32	SD422	*E. coli*	2020	Liaocheng	Chicken	+	R
33	HZ10A-1	*E. coli*	2024	Heze	Sewage	+	R
34	HZ8B-1	*E. coli*	2024	Heze	Sewage	+	R
35	DZC1-1	*E. coli*	2024	Dezhou	Sewage	+	0.93
36	DZC10-1	*E. coli*	2024	Dezhou	Sewage	+	0.97
37	HZ12A-1	*E. coli*	2024	Heze	Sewage	+	R
38	HZ5C-1	*E. coli*	2024	Heze	Sewage	+	R
39	HZ7C	*E. coli*	2024	Heze	Sewage	+	R
40	SD295	*E. coli*	2019	Qingdao	Chicken	−	
41	SD277	*E. coli*	2019	Qingdao	Chicken	−	
42	SD410	*E. coli*	2020	Liaocheng	Chicken	−	
43	SD77	*E. coli*	2018	Yantai	Duck	−	
44	SD44	*E. coli*	2018	Liaocheng	Chicken	−	
45	SD48	*E. coli*	2018	Liaocheng	Chicken	−	
46	SD302	*E. coli*	2019	Dongying	Chicken	−	
47	SD151	*E. coli*	2018	Weifang	Chicken	−	
48	SD47	*E. coli*	2018	Liaocheng	Chicken	−	
49	SD66	*E. coli*	2018	Yantai	Duck	−	
50	SD16	*E. coli*	2018	Liaocheng	Chicken	−	
51	SD41	*E. coli*	2018	Liaocheng	Chicken	−	
52	SD132	*E. coli*	2019	Liaocheng	Chicken	−	
53	SD275	*E. coli*	2019	Qingdao	Chicken	−	
54	SD158	*E. coli*	2019	Weifang	Chicken	−	
55	SD146	*E. coli*	2019	Liaocheng	Chicken	−	
56	SD372	*E. coli*	2020	Weifang	Chicken	−	
57	SD395	*E. coli*	2020	Weifang	Chicken	−	
58	SD338	*E. coli*	2019	Dongying	Chicken	−	
59	SD106	*E. coli*	2019	Liaocheng	Chicken	−	
60	SD394	*E. coli*	2020	Weifang	Chicken	−	
61	SD214	*E. coli*	2019	Yantai	Duck	−	
62	SD105	*E. coli*	2019	Liaocheng	Chicken	−	
63	SD150	*E. coli*	2019	Liaocheng	Chicken	−	
64	SD435	*E. coli*	2020	Liaocheng	Chicken	−	
65	SD82	*E. coli*	2018	Yantai	Duck	−	
66	SD390	*E. coli*	2020	Weifang	Chicken	−	
67	SD36	*E. coli*	2018	Liaocheng	Chicken	−	
68	SD27	*E. coli*	2018	Liaocheng	Chicken	−	
69	SD297	*E. coli*	2019	Qingdao	Chicken	−	
70	SD400	*E. coli*	2020	Weifang	Chicken	−	
71	SD171	*E. coli*	2019	Weifang	Chicken	−	
72	SD38	*E. coli*	2018	Liaocheng	Chicken	−	
73	SD192	*E. coli*	2019	Weifang	Chicken	−	
74	SD237	*E. coli*	2019	Yantai	Duck	−	
75	SD174	*E. coli*	2019	Weifang	Chicken	−	
76	SD172	*E. coli*	2019	Weifang	Chicken	−	
77	SD228	*E. coli*	2019	Yantai	Duck	−	
78	SD431	*E. coli*	2020	Liaocheng	Chicken	−	
79	SD403	*E. coli*	2020	Liaocheng	Chicken	−	
80	SD57	*E. coli*	2018	Yantai	Duck	−	
81	SD365	*E. coli*	2020	Weifang	Chicken	−	
82	SD364	*E. coli*	2020	Weifang	Chicken	−	
83	SD367	*E. coli*	2020	Weifang	Chicken	−	
84	SD366	*E. coli*	2020	Weifang	Chicken	−	
86	SD311	*E. coli*	2019	Dongying	Chicken	−	
87	SD298	*E. coli*	2019	Qingdao	Chicken	−	
88	SD137	*E. coli*	2019	Liaocheng	Chicken	−	
89	SD299	*E. coli*	2019	Qingdao	Chicken	−	
90	SD276	*E. coli*	2019	Qingdao	Chicken	−	
91	SD379	*E. coli*	2020	Weifang	Chicken	−	
92	SD310	*E. coli*	2019	Dongying	Chicken	−	
93	SD397	*E. coli*	2020	Weifang	Chicken	−	
94	SD368	*E. coli*	2020	Weifang	Chicken	−	
95	SD309	*E. coli*	2019	Dongying	Chicken	−	
96	SD376	*E. coli*	2020	Weifang	Chicken	−	
97	HZ3C−1	*E. coli*	2024	Heze	Sewage	−	
98	DZC4-1	*E. coli*	2024	Dezhou	Sewage	−	
99	DZB9	*E. coli*	2024	Dezhou	Sewage	−	
100	HZ9B-1	*E. coli*	2024	Heze	Sewage	−	
101	YTC25	*E. coli*	2024	Yantai	Sewage	−	

“+” indicated that phage could lysis bacteria, “−” indicated no lysis. The values in the table correspond to the susceptibility of each bacterial strain. “R” indicates full resistance, with no productive infection observed.

The bacteriophage vB_EcoM_SD286 showed productive infection in only a subset of the tested *E. coli* strains, while the majority of isolates proved resistant (Table 1). High replication efficiency, defined as an EOP value ≥ 0.5, was observed on several strains, including *E. coli* SD288, SD350, SD324, DZC1-1, and DZC10-1. An additional strain, *E. coli* SD339, supported plaque formation with an EOP above 0.1, albeit with visibly fewer plaques.

Overall, vB_EcoM_SD286 displays a narrow host range, with productive infection confined to a limited number of susceptible hosts. Among the 39 strains tested, only six—besides the primary host strain—were effectively infected. The vast majority of strains exhibited complete resistance, yielding EOP values of zero. These results indicate that vB_EcoM_SD286 is a highly specific phage whose infectivity is restricted to a narrow set of host strains.

### 3.2 Biological characteristics of phage vB_EcoM_SD286

**FIGURE 2:**
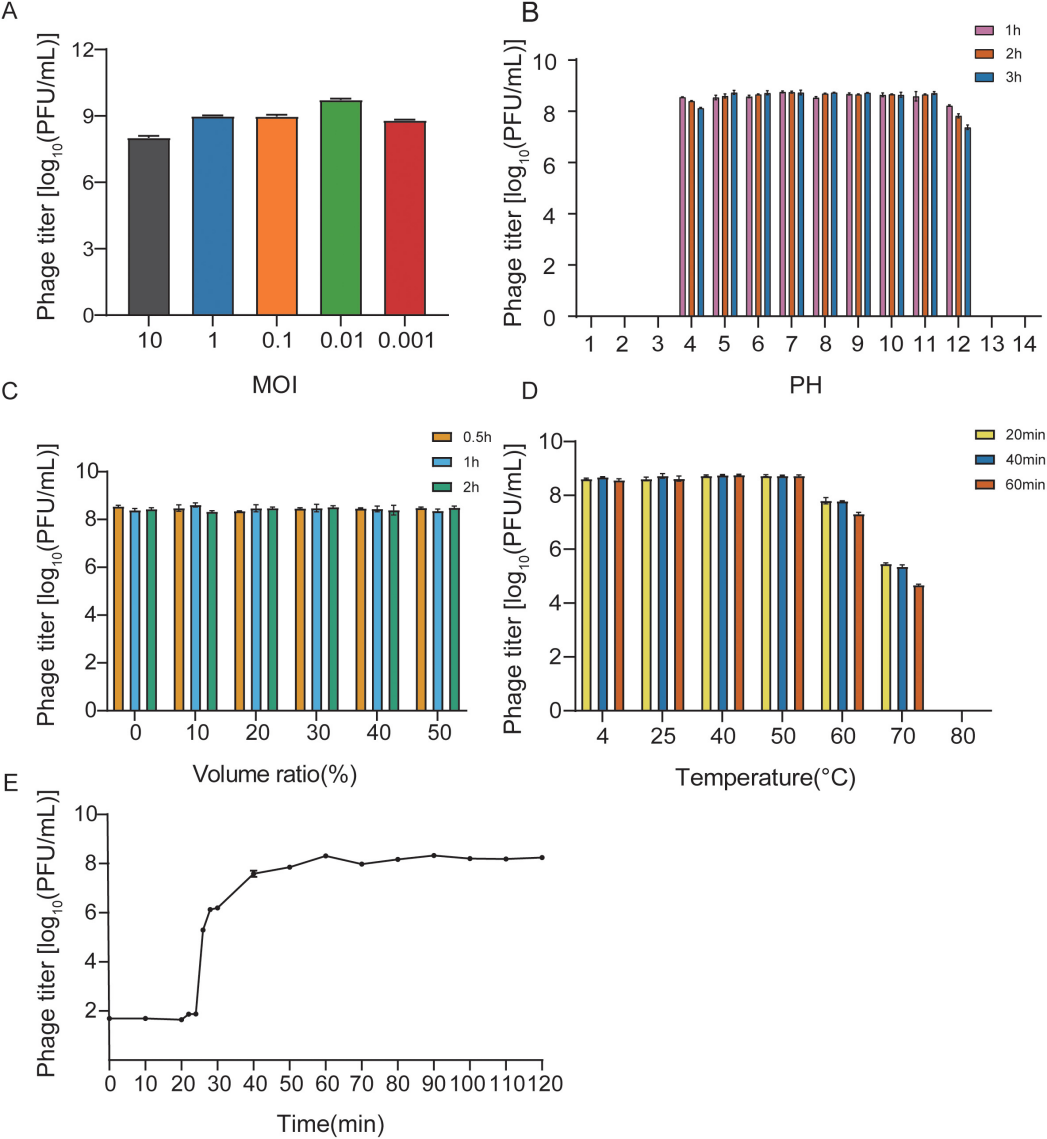
The biological characteristics of phage vB_EcoM_SD286. **(A)** Optimal multiplicity of infection of phage vB_EcoM_SD286. **(B)** The stability of phage vB_EcoM_SD286 in different pH value. **(C)** The stability of phage vB_EcoM_SD286 in different Chloroform volume ratio. **(D)** The stability of phage vB_EcoM_SD286 in different temperature. **(E)** One-step growth curve for phage vB_EcoM_SD286. The error bars represent the standard deviation (SD) of the mean.

At an MOI of 0.01, the titer of vB_EcoM_SD286 was highest (approximately 5.36 × 10^9^ PFU/mL), confirming this as the optimal MOI (Figure 2A). The phage retained high titers across a broad pH range (5–11) for up to 180 min, though its activity declined in a time-dependent manner at pH 4 and pH 12. Exposure to pH 3 or pH 13 reduced the titer to undetectable levels within 1 h (Figure 2B). Treatment with chloroform at volume ratios ranging from 0 to 50% for 30, 60, or 120 min did not significantly affect phage viability, further confirming the absence of lipid components in its structure (Figure 2C). In temperature stability assays, vB_EcoM_SD286 remained stable after incubation at 4 °C, 25 °C, and 40–50 °C for 20, 40, or 60 min. A slight titer reduction was observed after 20 min at 60 °C. At 70 °C, the titer decreased sharply in a time- and temperature-dependent manner, and the phage was completely inactivated within 20 min at 80 °C (Figure 2D). A one-step growth curve revealed a latency period of approximately 25 min, with the titer increasing rapidly between 25 and 40 min. From 40 to 60 min, the titer increased gradually until reaching a plateau after 60 min, with a burst size of approximately 33 PFU/cell (Figure 2E).

### 3.3 Phage vB_EcoM_SD286 genome sequencing and comparative genomics analysis

**FIGURE 3:**
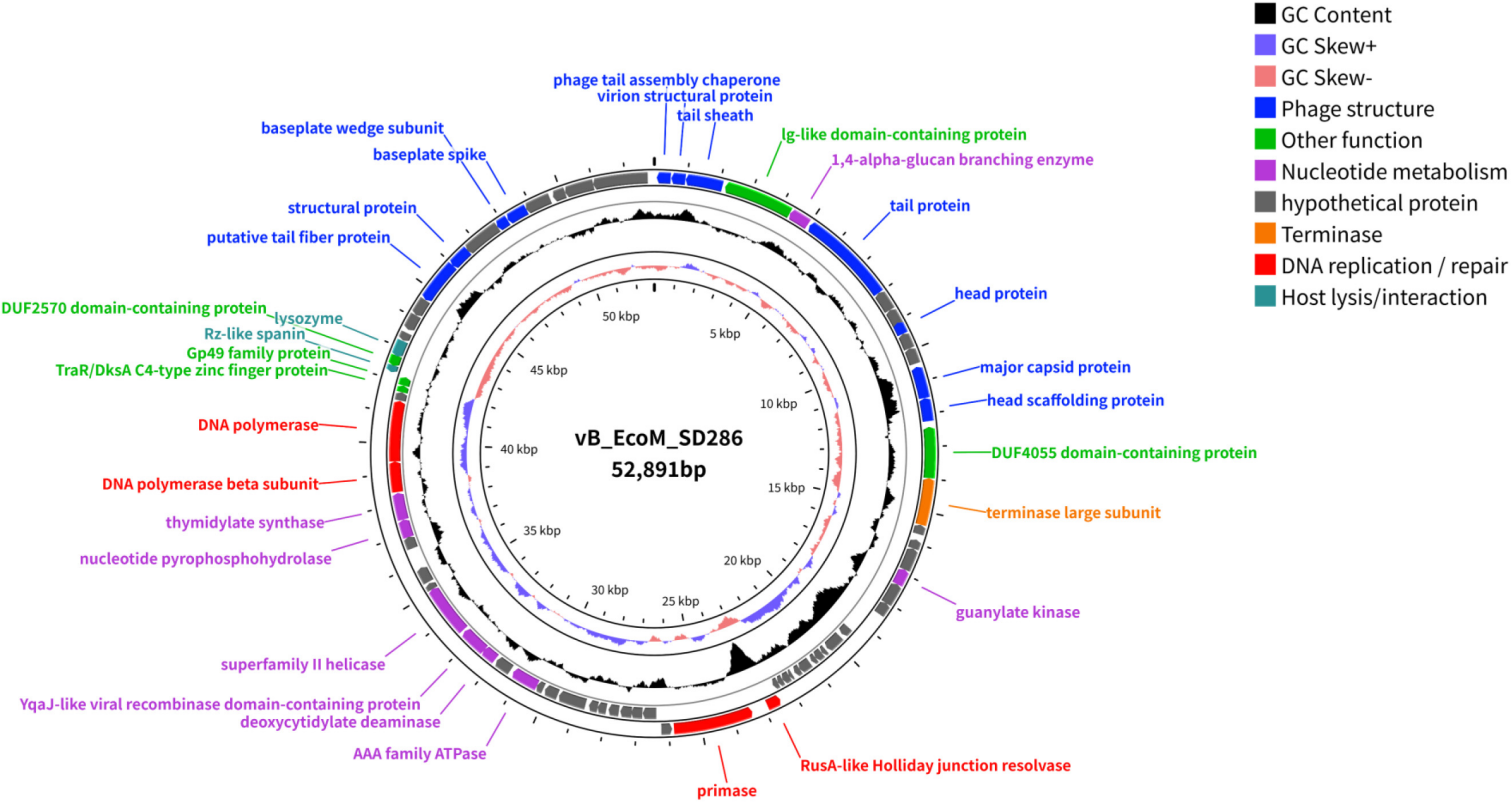
The whole genome map of phage vB_EcoM_SD286. ORFs are colored according to their proposed function: Phage structure—blue; Nucleotide metabolism—purple; hypothetical protein—gray; Terminase—orange; DNA replication/repair—red; Host lysis/interaction—dark green; other ORFs are colored with green.

The circular diagram of the phage vB_EcoM_SD286 genome is shown in Figure 3. Whole-genome sequencing revealed that phage vB_EcoM_SD286 is a linear double-stranded DNA molecule with a total length of 52,891 bp and a GC content of 46.06%. No tRNA genes, virulence factors, or antibiotic resistance genes were detected (Figure 3). We utilized PhaBOX (PhaTYP), a state-of-the-art deep learning tool, to analyze the genome. The analysis classified vB_EcoM_SD286 as “virulent phage” with high confidence. Genome annotation identified 74 open reading frames (ORFs), including 35 ORFs on the forward strand and 39 ORFs on the reverse strand. Among them, 31 ORFs encoded proteins with known functions, while 43 ORFs were predicted as hypothetical proteins (Table 2).

**TABLE 2:** Genomic annotation of *Escherichia* phage vB_EcoM_SD286.

CDS	Strand	Start	Stop	Type	Name
1	−	71	499	Phage structure	Phage tail assembly chaperone
2	–	518	949	Phage structure	Hypothetical protein
3	−	961	2100	Phage structure	Tail sheath
4	−	2187	4316	Hypothetical protein	
5	−	4328	4966	Nucleotide metabolism	1,4-alpha-glucan branching enzyme
6	−	5034	7991	Phage structure	Tail protein
7	−	7991	8614	Hypothetical protein Hypothetical protein	
8	−	8614	9141		
9	−	9065	9442	Phage structure	Head protein
10	−	9442	9930	Hypothetical protein Hypothetical protein	
11	−	9930	10430		
12	−	10535	11512	Phage structure	major capsid protein
13	−	11516	12229	Phage structure	Head scaffolding protein
14	−	12407	13969	Hypothetical protein	
15	−	13971	15419	Terminase	Terminase large subunit
16	−	15423	15713	Hypothetical protein Hypothetical protein Hypothetical protein	
17	−	15891	16181		
18	−	16181	16873		
19	−	16870	17376	Nucleotide metabolism	Guanylate kinase
20	−	17373	18047	Hypothetical protein Hypothetical protein Hypothetical protein Hypothetical protein Hypothetical protein Hypothetical protein Hypothetical protein Hypothetical protein Hypothetical protein Hypothetical protein Hypothetical protein Hypothetical protein Hypothetical protein	
21	−	18040	18441		
22	+	19373	19675		
23	+	19772	20341		
24	+	20406	20564		
25	+	20570	20827		
26	+	20824	20997		
27	+	21066	21455		
28	+	21455	21655		
29	+	21748	21873		
30	+	21863	22108		
31	+	22108	22287		
32	+	22284	22469		
33	−	22466	22897	DNA replication/repair	RusA-like Holliday junction resolvase
34	−	23394	25847	DNA replication/repair	Primase
35	−	25903	26235	Hypothetical protein Hypothetical protein Hypothetical protein DUF7173 domain-containing protein Hypothetical protein Hypothetical protein Hypothetical protein Hypothetical protein Hypothetical protein Hypothetical protein	
36	+	26389	26826		
37	+	26837	27205		
38*	+	27186	27557		
39	+	27602	27943		
40	+	28012	28302		
41	+	28299	28589		
42	+	28704	29609		
43	+	29658	30104		
44	+	30165	30395		
45	+	30395	31279	Nucleotide metabolism	AAA family ATPase
46	+	31387	31932	Hypothetical protein	
47	+	31994	32479	Nucleotide metabolism	Deoxycytidylate deaminase
48	+	32463	33341	Nucleotide metabolism	YqaJ-like viral recombinase domain-containing protein
49	+	33400	35070	Nucleotide metabolism	Superfamily II helicase
50	+	35057	35278	Hypothetical protein Hypothetical protein Hypothetical protein	
51	+	35325	35852		
52	+	36530	36928		
53	+	36915	37508	Nucleotide metabolism	Nucleotide pyrophosphohydrolase
54	+	37508	38392	Nucleotide metabolism	Thymidylate synthase
55	+	38423	39418	DNA replication/repair	DNA polymerase beta subunit
56	+	39418	41385	DNA replication/repair	DNA polymerase
57	+	41401	41676	Hypothetical protein Hypothetical protein Hypothetical protein	
58	+	41676	41900		
59	+	41900	42178		
60	−	42195	42413	Host lysis/interaction	Rz-like spanin
61	−	42397	42726	Hypothetical protein	
62	−	42705	43211	Host lysis/interaction	Lysozyme
63	−	43225	43485	Hypothetical protein Hypothetical protein Hypothetical protein	
64	−	43588	44091		
65	−	44095	44637		
66	−	44646	45980	Phage structure	Putative tail fiber protein
67	−	45973	46632	Phage structure	Hypothetical protein
68	−	46619	47791	Hypothetical protein	
69	−	47791	48162	Phage structure	Baseplate wedge subunit
70	−	48173	48820	Phage structure	Baseplate spike
71	−	48820	49617	Hypothetical protein Hypothetical protein Hypothetical protein Hypothetical protein	
72	−	49721	50113		
73	−	50113	51009		
74	−	51006	52688		

^“*”^ indicates the gene selected for functional investigation in this study.

### 3.4 Genetic characterization of phage vB_EcoM_SD286

**FIGURE 4:**
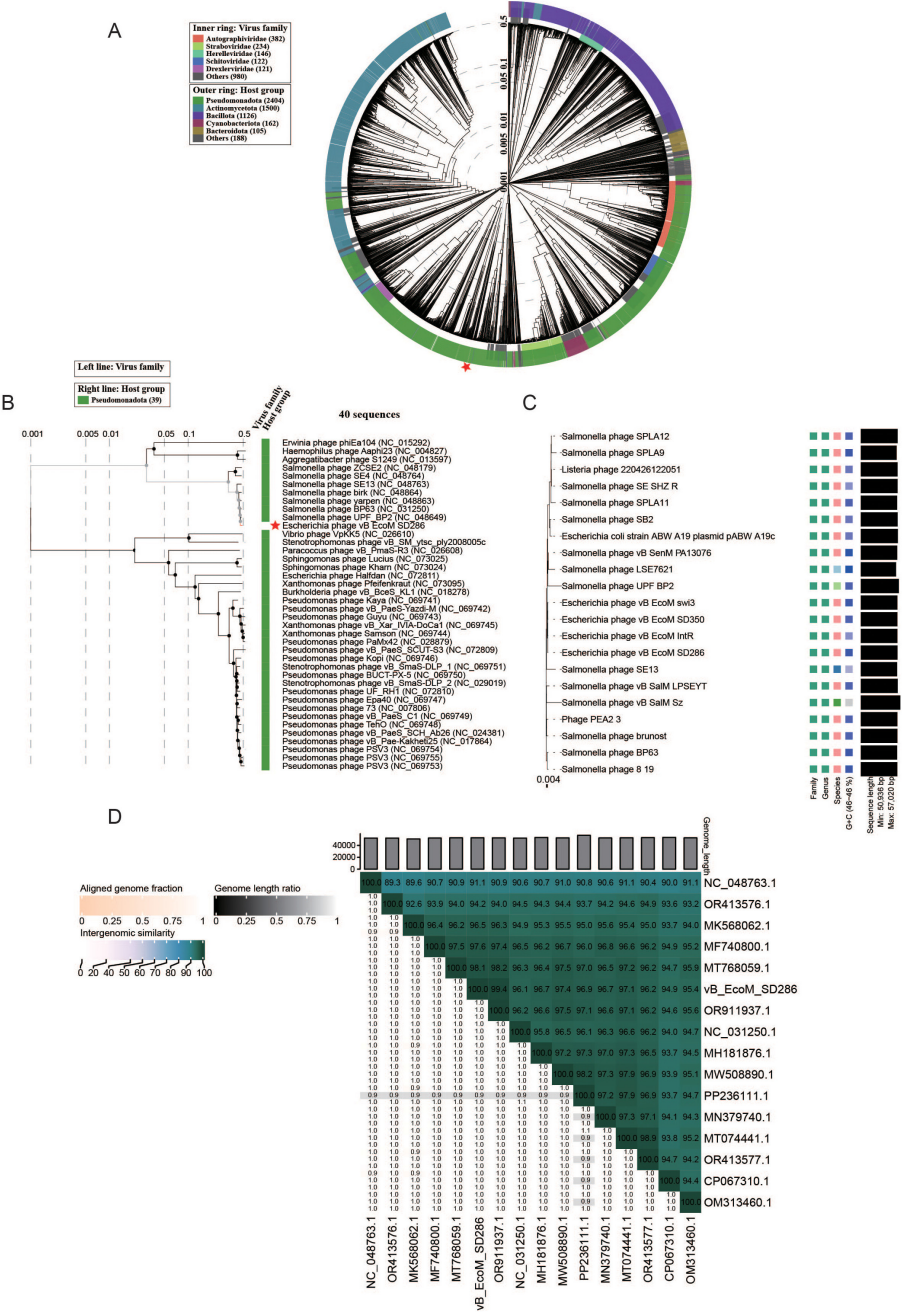
Genetic characterization of the phage vB_EcoM_SD286. **(A)** Based on the 5633 phages included in VipTree database, the proteomic tree was constructed for phage vB_EcoM_SD286 based on all-against-all genomic similarity scores. **(B)** The partial proteomic tree of phage vB_EcoM_SD286. **(C)** Phylogenetic analysis and classification of the phage vB_EcoM_SD286 genome via VICTOR. **(D)** The intergenomic similarity analysis of phage vB_EcoM_SD286 was used VIRIDIC. The “*” symbol denotes phage vB_EcoM_SD286.

Based on the 5633 phage genomes recorded in the Viptree database, vB_EcoM_SD286 was analyzed and an evolutionary tree was constructed using whole-genome similarity scores (SG) (Figure 4A). Thirty-nine phage sequences showing the highest similarity to vB_EcoM_SD286 were used to construct a local phylogenetic tree, excluding sequences with low or no similarity (Figure 4B). The analysis revealed that vB_EcoM_SD286 (PX226730.1) is most closely related to *Salmonella* phage UPF_BP2 (NC_048649), followed by *Salmonella* phage BP63 (NC_031250). Both UPF_BP2 and BP63 belong to the *Caudoviricetes* class and the *Rosemountvirus* genus. To further clarify the relationship between *Escherichia* phage vB_EcoM_SD286 and related phages, a Virus Classification and Tree Building (VICTOR) analysis was conducted based on whole-genome sequences (Figure 4C). The phylogenetic tree showed that vB_EcoM_SD286 clustered in the same branch as *Escherichia* phages vB_EcoM_IntR, vB_EcoM_swi3, and vB_EcoM_SD350. According to the International Committee on Taxonomy of Viruses (ICTV) classification system and phylogenetic analysis, vB_EcoM_SD286 was assigned to the *Caudoviricetes* class and *Rosemountvirus* genus.

Fifteen highly homologous phage genomes were extracted from NCBI and analyzed using VIRIDIC. As shown in Figure 4D, *Escherichia* phage vB_EcoM_SD286 exhibited high genomic similarity with all other strains (most exceeding 94%), especially with *Escherichia* phage vB_EcoM_SD350 (OR911937.1), showing 99.4% similarity, indicating an extremely close genetic relationship.

Further genomic structural analysis revealed that vB_EcoM_SD286 exhibited an aligned genome fraction close to 1.0 compared with the reference strain, along with highly consistent genome length ratios. These findings indicate that vB_EcoM_SD286 possesses a complete genomic structure, showing conservatism and high colinearity within this phage lineage.

### 3.5 Basic physical and chemical properties and structural analysis of the gp38 protein

**FIGURE 5:**
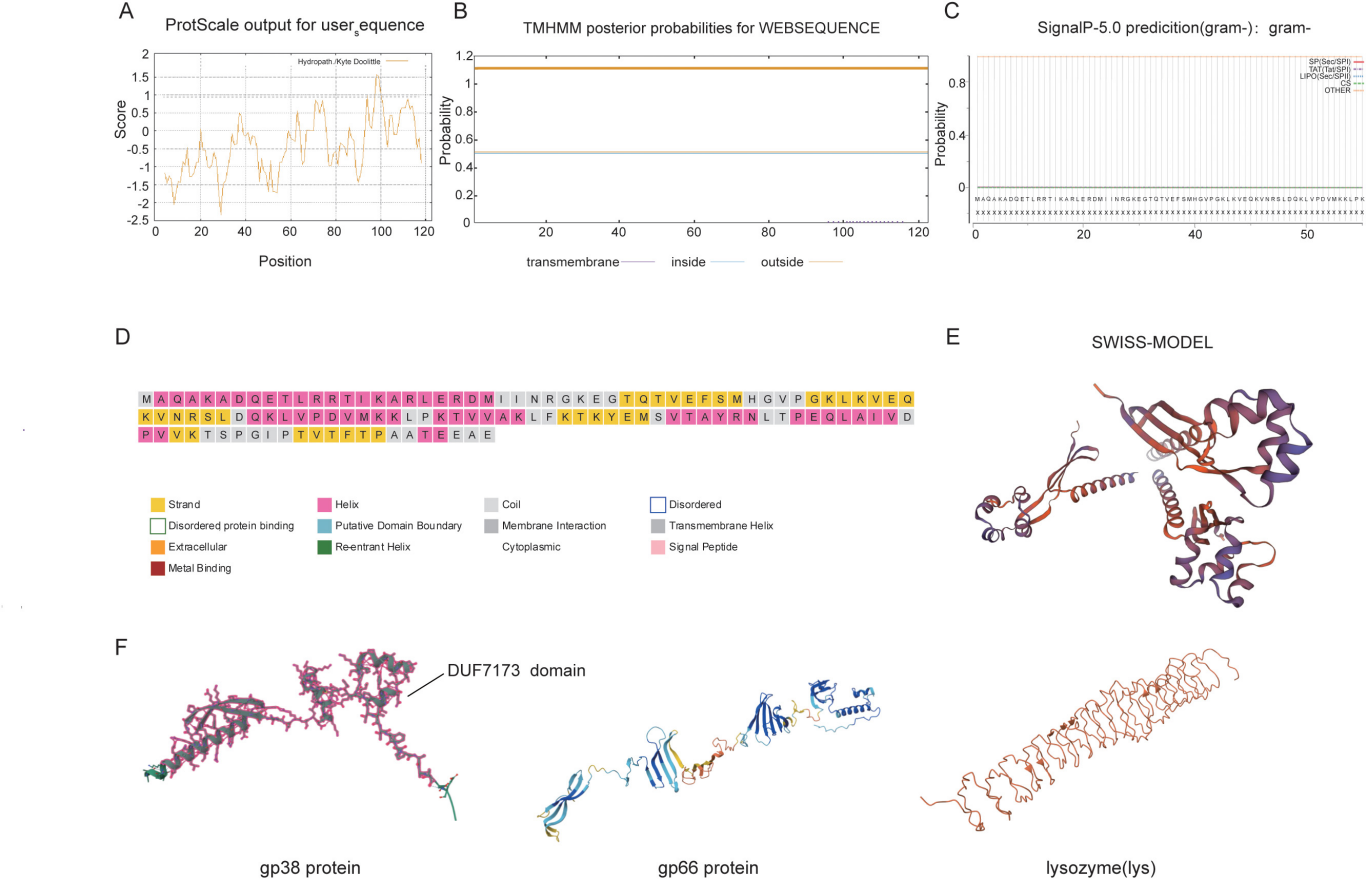
Basic physical and chemical properties and structural analysis of gp38 protein. **(A)** Hydrophilic and hydrophobic analysis of gp38 protein. **(B)** Transmembrance structure prediction of gp38 protein. **(C)** Prediction of signal peptides of gp38 protein. **(D)** Prediction of the secondary structure of gp38 protein. **(E)** Prediction of the tertiary structure of gp38 protein. **(F)** Structural models of gp38, gp66, and lysozyme, highlighting the location of the DUF domain in gp38.

Using the ProtParam server to predict and analyze the physicochemical properties of gp38, it was determined that the protein contains 123 amino acids, with a molecular mass of 13.75306 kDa, a theoretical isoelectric point (pI) of 9.65, and a half-life of > 10 h in *E. coli*. The instability index was 33.98, indicating that gp38 is a stable protein. The ProtScale server was used to predict the hydrophilicity and hydrophobicity of gp38 (Figure 5A). Most amino acid residues were found in hydrophilic regions, indicating that gp38 is a stable hydrophilic protein. Using the TMHMM Server v2.0 and Novopro Server, the transmembrane domains and signal peptides of gp38 were predicted. TMHMM analysis showed no transmembrane helices, with a 49.6% probability that the N-terminus is located within the membrane and no significant membrane localization tendency (Figure 5B). The entire sequence was predicted to be located outside the membrane. The probability of a signal peptide was 0.379%, far below the 5% threshold, clearly indicating the absence of a signal peptide (Figure 5C). Secondary structure prediction using PSIPRED showed that α-helices, β-sheets, and random coils accounted for 44.7, 28.5, and 26.8%, respectively (Figure 5D). Tertiary structure prediction using SWISS-MODEL indicated that gp38 exhibits an irregular overall shape (Figure 5E). Furthermore, the three-dimensional structures of the three relevant proteins—gp38, gp66, and lysozyme(lys)—were predicted using AlphaFold3 and are presented in Figure 5F. Based on analysis of the three-dimensional structure, the gp38 protein predominantly exists as a monomer. In the structural model of gp38, the location of the DUF domain (residues 6–119) is explicitly indicated.

### 3.6 Expression and purification of the gp38 protein

**FIGURE 6:**
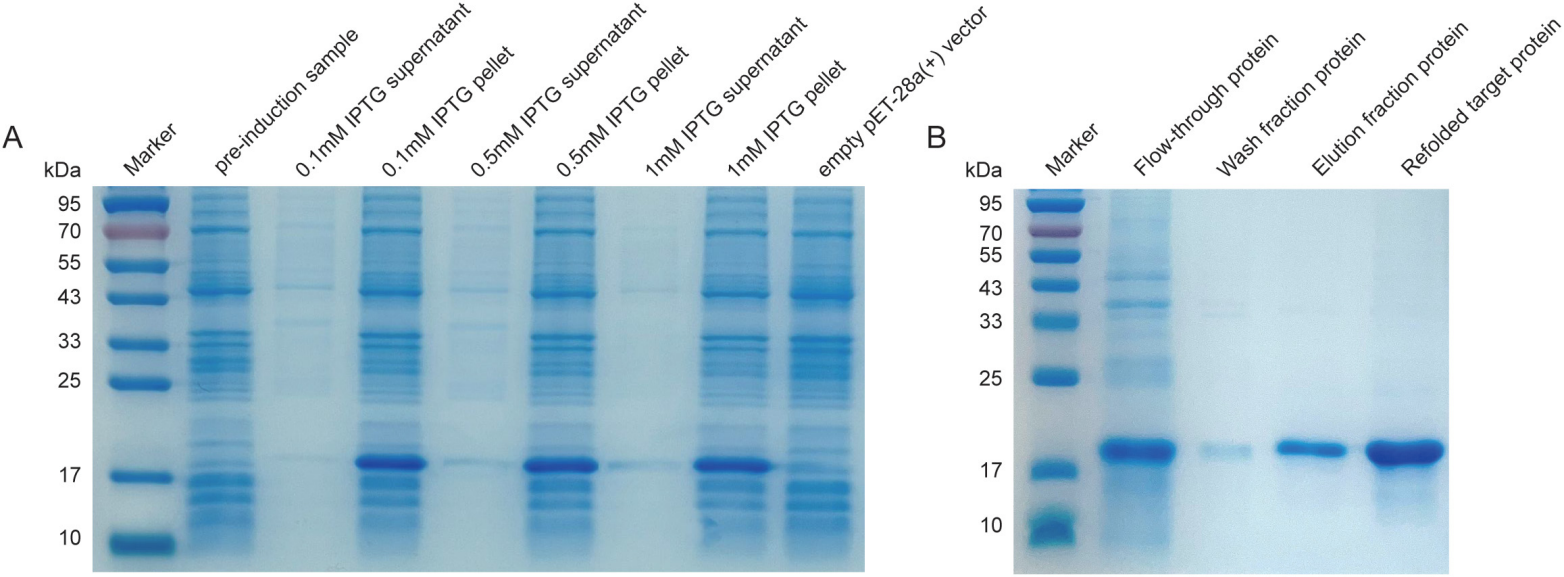
Expression and purification of gp38 protein. **(A)** Evaluation of optimal conditions for gp38 protein on SDS-PAGE: all conditions were conducted at 37°C. **(B)** Purification and renaturation of gp38 protein. The washing buffer contained 50 mM imidazole; The elution buffer contained 250 mM imidazole.

The gp38 fragment was amplified by PCR from the vB_EcoM_SD286 genome using specific primers. The gp38 gene fragment was inserted into the *Bam*HI and *Xho*I sites to construct the recombinant plasmid pET-28a(+)-gp38, which was then expressed in the *E. coli* BL21(DE3) strain, yielding BL21-pET-28a(+)-gp38. The expression strain was induced with IPTG, and the cells were sonicated to release the target protein. SDS–PAGE analysis revealed that gp38 was clearly expressed in the precipitate fractions at different IPTG concentrations (Figure 6A). Therefore, 37°C and 0.5 mmol/L IPTG were selected as the optimal induction conditions for subsequent experiments.

After large-scale induction, inclusion bodies were collected and subjected to Ni–NTA affinity column purification. The target protein fraction eluted with imidazole was analyzed by SDS–PAGE, confirming high purification efficiency. The purified protein was then refolded through multi-step urea gradient dialysis renaturation. SDS–PAGE analysis of samples before and after denaturation and renaturation confirmed successful inclusion body protein refolding (Figure 6B). The concentration of the refolded gp38 protein was measured using the Bradford protein assay kit, yielding a final concentration of 1.15 mg/mL.

The control proteins gp66 and lys were expressed, purified, and refolded using an identical experimental workflow. All proteins were subsequently prepared for functional assays.

### 3.7 gp38 protein inhibits phage vB_EcoM_SD286 adsorption to host bacteria

**FIGURE 7:**
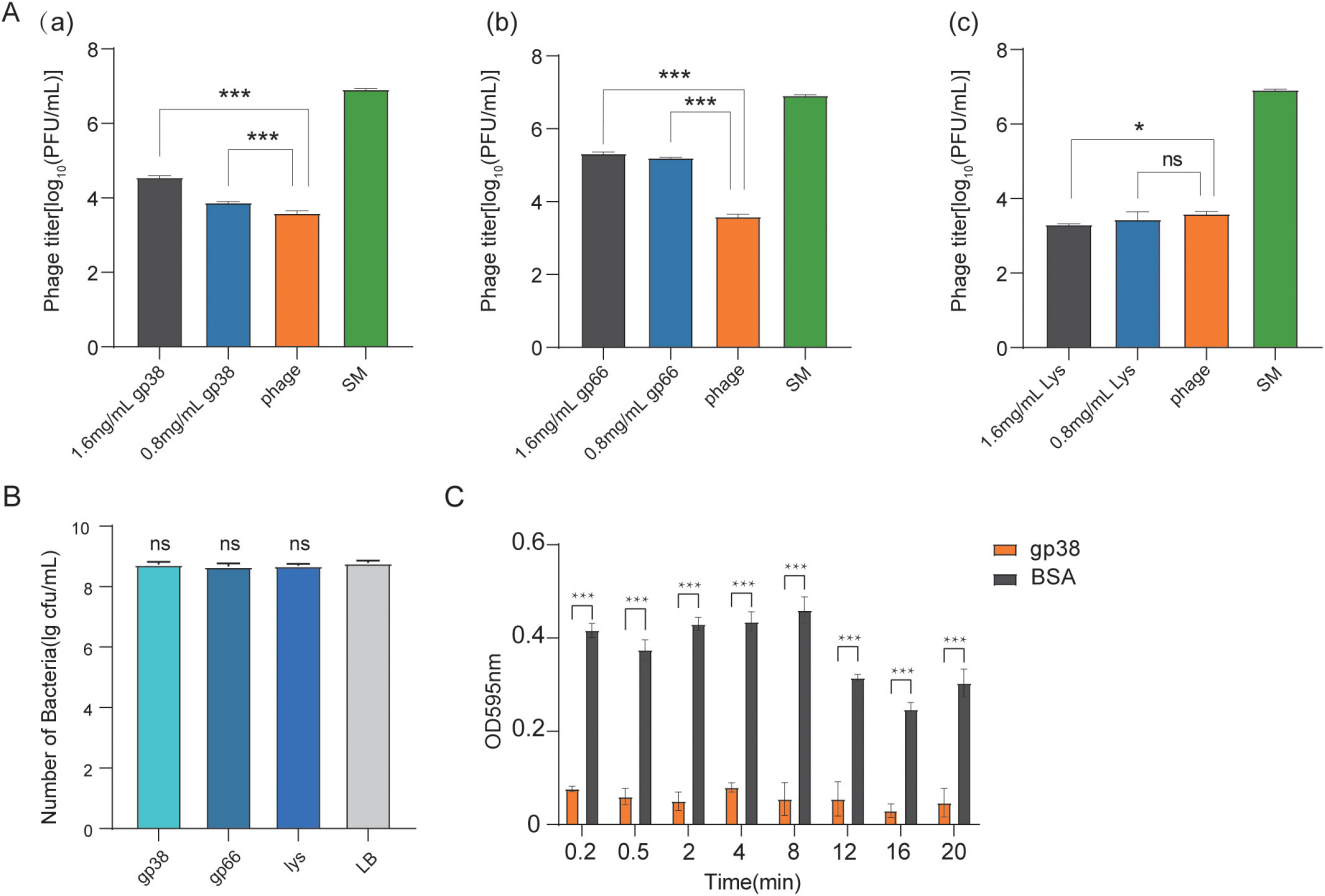
The effect of gp38 protein on the adsorption of phage vB_EcoM_SD286. **(A)** Free phage titer of vB_EcoM_SD286 determined after incubation with different concentrations of recombinant proteins (gp38, gp66, and Lys proteins) and a control group (SM buffer). (a) Competitive adsorption test of protein gp38 and vB_EcoM_SD286. (b) Competitive adsorption test of protein gp66 (positive control) and vB_EcoM_SD286. (c) Competitive adsorption test of protein Lys (negative control) and vB_EcoM_SD286. **(B)** Assessment of bacteriolytic activity of recombinant gp38, gp66, and Lys proteins. **(C)** The binding capacity of gp38 and BSA (control group) to the host bacterium was assessed at different time points by measuring the OD595 value. This result represents a quantitative analysis after correction for the negative control (PBS blank group). Statistical significance notation: *p < 0.05, **p < 0.01, ***p < 0.001; ns, not statistically significant. Error bars denote mean ± standard error.

To validate the effect of recombinant gp38 protein on the adsorption of phage vB_EcoM_SD286 to host bacteria, competitive adsorption experiments were conducted to evaluate its inhibitory effect by measuring free phage titers. The results showed that recombinant gp38 protein significantly inhibited the adsorption of phage vB_EcoM_SD286. As shown in Figure 7A, at a gp38 concentration of 1.6 mg/mL, the free phage titer was significantly higher than that of the negative control group (without added competitor), indicating that gp38 competitively occupied receptor sites on the surface of *E. coli* SD405, thereby blocking normal phage adsorption. In contrast, no competitive inhibition was observed in the lysozyme (Lys) control group (Figure 7c), ruling out non-specific interference and confirming the specific competitive adsorption activity of gp38.

The inhibitory effects of two gp38 protein concentrations (1.6 and 0.8 mg/mL) were also compared. Results showed that 1.6 mg/mL gp38 produced a more pronounced competitive inhibition (Figure 7a), suggesting a concentration-dependent mechanism. Additionally, the gp66 protein, used as another control (Figure 7b), did not exhibit significant competitive inhibition, further supporting the specificity of gp38 function.

To rule out the possibility that the observed inhibition of phage adsorption resulted from bacteriolytic activity, the effect of gp38 on bacterial viability was assessed. As shown in Figure 7B, treatment with gp38 did not reduce the CFU/mL of *E. coli* SD405 compared to the untreated control, confirming that gp38 lacks bacteriolytic properties under the experimental conditions. These results collectively demonstrate that gp38 specifically inhibits phage adsorption through a receptor-blocking mechanism, independent of host cell lysis.

To further validate this effect, the binding capacity of the gp38 protein to host bacteria was evaluated. Quantitative analysis, calibrated against a negative control (PBS + gp38), confirmed a highly specific, rapid, and efficient binding interaction between gp38 and the host bacteria. As shown in Figure 7C, the binding amount between gp38 and the bacteria stabilized within 2–4 min, likely reaching saturation, and remained stable beyond 16 min without significant variation. After correction by the negative control, the BSA group effectively excluded non-specific binding, confirming that the data accurately reflected true protein–bacterium interactions. These findings indicate that the gp38 protein functions as a critical bacterial receptor-binding component, with a rapid and specific binding process completing within minutes. Collectively, the results suggest that gp38 exhibits RBP-like functionality and is likely the receptor-binding protein of phage vB_EcoM_SD286.

## 4 Discussion

This study focuses on addressing the ongoing challenges posed by APEC, a significant pathogen in poultry. The diversity of APEC serotypes and antigenic variability severely limit the efficacy of traditional vaccines. Although antibiotics have been widely and effectively used, this practice has inadvertently driven the emergence of multidrug-resistant strains, creating a dual threat to both poultry health and food safety. Against this backdrop, it is particularly urgent to develop new antimicrobial strategies, such as phage therapy, to combat multidrug-resistant bacterial infections (Zhang and Cheng, 2022).

Phage therapy has attracted considerable attention because of its advantages, including high host specificity, minimal interference with intestinal microecology, and a mechanism of action fundamentally different from that of antibiotics, as it utilizes bacteria to infect, replicate, and lyse bacterial cells (Thakali et al., 2024). However, a major bottleneck in its clinical application lies in its narrow host range (de Jonge et al., 2019). This specificity primarily stems from the precise molecular recognition and binding between phage RBPs and host bacterial surface receptors (such as lipopolysaccharides (LPS) and outer membrane proteins) during the early stages of infection (Ding et al., 2024). As demonstrated by the T4 bacteriophage, which specifically recognizes OmpC via the gp37 protein, RBPs act as the “keys” determining the bacteriophage infection spectrum (Suga et al., 2021). Therefore, identifying and understanding the RBPs of novel bacteriophages and their interaction mechanisms constitute the core foundation for expanding bacteriophage host spectra and designing more effective phage cocktail formulations (Peng et al., 2025).

In this study, we utilized the *E. coli* tailed bacteriophage vB_EcoM_SD286, isolated from a farm in Shandong Province, to explore its infection mechanism in depth. A systematic research strategy was adopted: first, we revealed the biological characteristics and genetic information of the bacteriophage through biological property analysis and whole-genome sequencing; The efficiency of plating (EOP) profiles of phages vB_EcoM_SD286 and vB_EcoS-TPF103dw both demonstrate narrow host specificity (Campos et al., 2025). Productive infection (EOP ≥ 0.5) for vB_EcoM_SD286 was limited to only 6 of 39 tested *E. coli* strains. Similarly, vB_EcoS-TPF103dw infected primarily *E. coli* O103 and some O26 isolates, with no activity against other STEC serogroups, *Salmonella*, or *Listeria*.

This specificity limits broad-spectrum activity but offers advantages in targeted biocontrol. vB_EcoS-TPF103dw could be applied where O103/O26 are predominant, while vB_EcoM_SD286 could target its specific hosts. However, for mixed-species or multi-serovar biofilms, both phages would likely require formulation into cocktails with other phages or sanitizers to broaden coverage and mitigate resistance. Notably, EOP varied even among susceptible strains. Some O103 isolates showed high EOP (> 0.9) with vB_EcoS-TPF103dw, while others had moderate efficiency (> 0.1)—a pattern also seen with vB_EcoM_SD286. This highlights that phage efficacy can differ substantially within a serogroup, emphasizing the need for strain-level validation before application.

In summary, these phages illustrate the trade-off between precision and breadth. Their narrow host range allows targeted control but necessitates combinatorial strategies for effective biofilm disruption in complex communities. Future work should clarify their molecular mechanisms of host recognition and depolymerase activity, and evaluate their synergy with other antimicrobials.

The genome of vB_EcoM_SD286 was analyzed with PhaBOX (PhaTYP) (Shang et al., 2022), a state-of-the-art deep-learning tool for predicting phage lifestyle. The analysis confidently classified vB_EcoM_SD286 as a virulent (lytic) phage. This bioinformatic assignment is consistent with its observed phenotypic behavior—notably efficient host lysis—and with the absence of lysogeny-associated genes (e.g., integrase, repressor) in its genome. Such a classification further supports the potential of vB_EcoM_SD286 as a targeted antimicrobial agent, aligning well with its previously described narrow host range. Then, through comparative genomic analysis combined with bioinformatics investigation of key proteins, we successfully obtained recombinant proteins using prokaryotic expression technology. The most critical step involved the innovative application of competitive adsorption experiments for functional validation. By constructing recombinant protein expression vectors and inducing target protein expression in host bacteria, we were able to assess the impact of recombinant proteins on the adsorption ability of phage vB_EcoM_SD286 toward its host bacteria. If a recombinant protein competes with the bacteriophage for binding to the same receptor sites on the host surface, it is expected to significantly reduce the bacteriophage adsorption rate. This *in vitro* approach provides a powerful and relatively simple tool for directly measuring specific binding interactions between the recombinant protein and host bacterial surface receptors. Successful screening and validation of novel receptor-binding proteins would not only provide a molecular basis for understanding the infection specificity of vB_EcoM_SD286 but also lay the groundwork for identifying the specific receptor types recognized by bacteriophages, such as outer membrane proteins or LPS structures. Ding et al. reported, through ELISA assays, that the purified orf11 protein binds specifically to LPS from *M. aerodenitrificans* strain LH9, supporting the hypothesis that the protein interacts with this LPS (Ding et al., 2024). Similarly, in a functional characterization of an RBP, Ge et al. identified and confirmed that the tail fiber protein Lp35 acts as the RBP of phage LP31, as demonstrated by its ability to competitively inhibit phage adsorption through specific binding to host LPS (Ge et al., 2023).

To infer the potential function of the gp38 protein, a BLASTP search was performed against the UniProtKB database (The UniProt Consortium, 2024). The analysis revealed that its closest homologs are annotated as “DUF7173 domain-containing proteins,” suggesting that gp38 is a putative member of this family. Domains of Unknown Function (DUFs) represent large protein families that contain at least one conserved domain without characterized biological roles. In plants, numerous DUF families have been implicated in diverse biological processes. As reviewed by Luo et al., studies on plant DUFs have provided valuable insights into their functions and potential roles (Luo et al., 2024). Moreover, functional investigations of specific DUF families have yielded notable discoveries. For instance, Hou et al. demonstrated that DUF221-containing proteins constitute a novel family of osmosensitive calcium-permeable cation channels conserved across eukaryotes (Hou et al., 2014). Emerging evidence further indicates that DUFs also play key roles in microbe-phage interactions. For example, recent work on the HerA-DUF bacterial defense complex revealed that DUF domains can act as critical switches or sensory modules in the response to phage infection, underscoring their functional plasticity and importance in molecular dialogue (Rish et al., 2025). However, the functions of such proteins in non-plant systems, including microorganisms, remain poorly understood, particularly because the biological role of the DUF7173 domain has not yet been elucidated. Therefore, this study aims to explore the potential functions of the DUF7173 domain in a microbial context, with the gp38 protein serving as the primary subject of analysis.

This study confirmed both the efficiency and specificity of gp38 as a host bacterial receptor through detailed binding kinetics and specificity analyses, thereby providing a solid foundation for understanding its molecular interaction mechanisms. These findings offer important molecular evidence for investigating pathogen–host interaction processes. The identification of binding sites establishes a direction for further in-depth research. The competitive adsorption assays clearly demonstrated that the recombinant gp38 protein can interfere with phage adsorption, implying that both molecules compete for identical or closely related receptor sites on the host cell surface.

The hypothetical protein described in this study was originally predicted by genome sequencing with a known coding sequence but without verified biological functions such as structural, enzymatic, or binding activity. Through experimental validation, this work confirmed that the hypothetical gp38 protein engages in competitive adsorption with bacteriophages, highlighting a key direction for elucidating host specificity mechanisms in phages. Nevertheless, this study has several limitations that should be acknowledged. First, although competitive adsorption assays confirmed the receptor-binding function of gp38, the specific molecular identity of its corresponding receptor on the host bacterial surface—such as a particular outer membrane protein or LPS component—remains to be elucidated (Islam et al., 2019). Second, the exact functional domains within gp38 responsible for receptor recognition have not been mapped. Third, while effective, the *in vitro* competitive adsorption assay does not fully capture the complexity of the *in vivo* environment, where additional factors could influence binding efficacy. Finally, the generalizability of our findings to other APEC serotypes or *E. coli* pathotypes requires further validation using a broader panel of bacterial strains.

This study preliminarily identified potential RBP candidates, although precise mapping of their functional domains has not yet been performed. Determining the critical structural motifs or amino acid residues responsible for receptor binding is essential for clarifying recognition specificity and for engineering modified RBPs with enhanced characteristics, including a wider host range or stronger binding affinity. Future work could focus on dissecting functional regions within RBPs through site-directed mutagenesis, domain-swapping analyses, or directed evolution experiments, thereby deepening our understanding of RBP function (Liu et al., 2020). Meanwhile, the ongoing expansion of genomic sequencing across diverse species is rapidly increasing the catalog of Domains of Unknown Function (DUFs). Although characterizing these DUFs remains a substantial challenge, they represent a valuable repository of unexplored genes. Integrating bioinformatics, multi-omics approaches, and experimental validation will be crucial for uncovering their biological significance, ultimately leading to a deeper comprehension of complex cellular mechanisms.

In summary, this study successfully established a comprehensive workflow for screening and validating phage receptor-binding proteins by integrating genomic analysis, bioinformatics prediction, molecular biology techniques, and functional validation assays. The recombinant proteins identified from phage vB_EcoM_SD286, which exhibit receptor-binding protein-like activity, not only enhance understanding of the specific phage–host interaction mechanisms but also provide valuable molecular targets and a theoretical framework for the design of broad-spectrum or improved phage formulations based on RBP engineering. For instance, understanding the RBPs and host spectra of multiple phages can facilitate the rational design of phage combinations capable of covering a broader range of APEC serotypes. Moreover, the potential of recombinant RBPs as novel antimicrobial agents or diagnostic tools could be a promising direction for future research. While translating these findings into clinical or field applications faces challenges such as *in vivo* delivery efficiency and host immune response management, this study establishes a strong molecular foundation for developing next-generation phage therapies against APEC infections and other bacterial diseases.

## 5 Conclusion

This study successfully isolated and characterized vB_EcoM_SD286, a strictly lytic phage with a narrow but efficient infectivity profile against specific *E. coli* strains. Its defined host range and virulent lifestyle were confirmed through biological and bioinformatics analyses. Furthermore, between the recombinant gp38 protein and the host bacterium confirmed that gp38 exhibits strong adsorption activity toward the host, functioning similarly to an RBP. This discovery provides a solid molecular foundation for the further identification and characterization of novel RBPs in *E. coli* phages and offers valuable insights for the development of phage-based antibacterial strategies.

## Data Availability

The datasets presented in this study can be found in online repositories. The names of the repository/repositories and accession number(s) can be found in the article/supplementary material.

## Footnotes

1
http://www.ncbi.nlm.nih.gov/BLAST

2
https://lowelab.ucsc.edu/tRNAscan-SE/index.html

3
http://www.mgc.ac.cn/cgi-bin/VFs/v5/main.cgi

4
https://card.mcmaster.ca/analyze/rgi

5
https://www.genome.jp/viptree/upload

6
https://ggdc.dsmz.de/victor.php

7
https://rhea.icbm.uni-oldenburg.de/viridic/

8
https://web.expasy.org/protparam/

9
https://web.expasy.org/protscale/

10
https://services.healthtech.dtu.dk/services/TMHMM-2.0/

11
https://novopro.cn/tools/signalp

12
http://bioinf.cs.ucl.ac.uk/psipred/

13
https://swissmodel.expasy.org/

14
https://alphafoldserver.com/
